# Soil pH mediates the balance between stochastic and deterministic assembly of bacteria

**DOI:** 10.1038/s41396-018-0082-4

**Published:** 2018-03-07

**Authors:** Binu M. Tripathi, James C. Stegen, Mincheol Kim, Ke Dong, Jonathan M. Adams, Yoo Kyung Lee

**Affiliations:** 1 Korea Polar Research Institute, Incheon, 21990 Republic of Korea; 2 Pacific Northwest National Laboratory, 902 Battelle Boulevard, P.O. Box 999, Richland, WA 99352 USA; 3 Department of Biological Sciences, College of Natural Sciences, Seoul National University, 1 Gwanak-ro, Gwanak-gu, Seoul, 08826 Republic of Korea; 4 School of Water, Energy and Environment, Cranfield University, Cranfield, MK43 0AL UK

**Keywords:** Microbial ecology, Biodiversity

## Abstract

Little is known about the factors affecting the relative influences of stochastic and deterministic processes that govern the assembly of microbial communities in successional soils. Here, we conducted a meta-analysis of bacterial communities using six different successional soil datasets distributed across different regions. Different relationships between pH and successional age across these datasets allowed us to separate the influences of successional age (i.e., time) from soil pH. We found that extreme acidic or alkaline pH conditions lead to assembly of phylogenetically more clustered bacterial communities through deterministic processes, whereas pH conditions close to neutral lead to phylogenetically less clustered bacterial communities with more stochasticity. We suggest that the influence of pH, rather than successional age, is the main driving force in producing trends in phylogenetic assembly of bacteria, and that pH also influences the relative balance of stochastic and deterministic processes along successional soils. Given that pH had a much stronger association with community assembly than did successional age, we evaluated whether the inferred influence of pH was maintained when studying globally distributed samples collected without regard for successional age. This dataset confirmed the strong influence of pH, suggesting that the influence of soil pH on community assembly processes occurs globally. Extreme pH conditions likely exert more stringent limits on survival and fitness, imposing strong selective pressures through ecological and evolutionary time. Taken together, these findings suggest that the degree to which stochastic vs. deterministic processes shape soil bacterial community assembly is a consequence of soil pH rather than successional age.

## Introduction

Understanding the fundamental ecological processes that shape the assembly of microbial communities is a major challenge in microbial ecology [1]. The assembly of microbial species in a local community is influenced by two types of ecological processes, namely deterministic and stochastic [2, 3]. Deterministic processes include ecological selection imposed by abiotic and biotic factors, which influence organismal fitness and thereby determine the composition and relative abundance of species [4, 5]. Stochastic processes, in contrast, involve random birth, death, and dispersal events that result in patterns of species composition indistinguishable from those produced by random chance alone [6, 7]. Both deterministic and stochastic processes act concurrently to regulate the assembly of ecological communities [8–11]. However, variation in strength of ecological selection and rates of dispersal influence the relative importance of deterministic and stochastic processes across time, space, and from one system to another [12–14].

An open question is the degree to which differences in the balance between stochastic and deterministic processes are driven by differences between environments rather than time itself. Successional soils represent a series of soils of different ages with varying abiotic and biotic characteristics [15]. The pedogenesis (soil development) processes along successional soils results in directional change in nutrient content and often, but not always, lead to declines in pH [16–19]. Over the past decade, several studies have characterized the shifts in soil microbial communities along successional soils—on both shorter [20–25] and longer [26, 27] time scales. However, relatively little is known about the underlying ecological processes that govern the assembly of microbial communities in successional soils [13, 21, 28]. One limitation is that in any single dataset or system, environmental conditions often vary systematically with time such that these variables are confounded. Bringing together a set of successional systems that differ in the relationship between soil age and environmental properties is ideal for disentangling the relative contributions of the environment vs. time.

Recent studies have indicated that in early successional soils, bacterial community assembly is largely governed by stochastic processes, with the relative importance of deterministic processes increasing progressively in later successional soils [13, 28]. However, it is still not clear what underlying environmental factors or community processes may be changing between early and late successional soils to produce this increased importance of determinism. For instance, it is well known that soil pH is very important in determining variation in bacterial community structure and diversity [29–33]. As pH often changes along successional soils [17], this could be the main cause of the observed trends in bacterial community assembly and also influence the relative importance of stochastic and deterministic processes across successional soils. Alternatively, time itself could be the driver wherein biological interactions change through time to alter the balance between stochastic and deterministic processes. To examine whether differences in soil pH (and other environmental variables) or time were the primary drivers of shifts in the stochastic-deterministic balance, we carried out a meta-analysis across a range of different successional soils, not all of which show the same trend in soil pH over time. This is a powerful design whereby successional age does not consistently co-vary with pH. Because successional age and pH do not confound each other, their relative influences can be evaluated. We applied an ecological null modeling approach to analyze the community assembly processes with the following questions:

1. Are there a predictable differences in bacterial community assembly across successional soils, regardless of differences in local environmental constraints? If community assembly processes are driven primarily by the amount of time a local community has had to develop, we expect ecological selection to be relatively weak during early succession and to increase in strength with time. In this case, the influence of stochastic processes relative to deterministic processes should decrease with successional age. On the other hand, if niches become more available as nutrients accumulate in later successional soils [17], a broader range of species may be able to establish. This scenario would lead to an increase in the relative influence of stochastic processes. In either case, there should be a directional shift in the balance between stochastic and deterministic processes regardless of differences in the abiotic environment.
2. Does soil pH explain differences in bacterial community assembly processes along successional soils, irrespective of successional age? An earlier conceptual model suggested that lack of strong environmental filtering in early successional soils leads to dominance of stochastic processes [13]. This suggests that time itself is not a driver of community assembly processes, and that assembly processes change through time because of changes to the abiotic environment. In this case, we expect that the balance between stochastic and deterministic processes will vary with pH (or other environmental variables) regardless of successional age.

## Materials and methods

### Datasets

For studying the bacterial community assembly and relative influence of ecological processes along successional soils, we compiled six datasets of varying soil pH gradients between early and late successional soils (Table 1). The four shorter-term (~150 years) datasets of successional soils were: Austre Lovénbreen Glacier (AL) [22], Midtre Lovénbreen Glacier (ML) (newly presented in this study), Damma Glacier (DM) [24], and Easton Glacier (ES) [23]. The two longer-term datasets of successional soils were 120,000-year-old Franz Josef Glacier (FJ) [26], and ~4000-year-old Wilderness Park sand-dune soil succession (SD) [27]. Except for the ML site, the sequence and metadata were obtained directly from authors or downloaded from the public repository. In the case of ML, soil samples were collected along the glacier chronosequence in summer 2014. A total of 39 samples were collected from three transects from the glacier terminus to the edge of the foreland moraine (for detailed information on sampling, see ref. [34]). The successional datasets showed a strong influence of pH and little influence of successional age. Given that pH had a much stronger association with community assembly processes than did successional age, we evaluated whether the inferred influence of pH was maintained in a broader range of soil ecosystems. To do so, we studied 130 globally distributed soil samples that were collected without regard for successional age [29, 31]. Like all soils, these have a successional history, but this information is not available in the global metadata, and they were not systematically collected with respect to successional stage. Hence, these samples represent a heterogeneous set of soils that differ in climate, vegetation, etc., and we assume they also differ in successional history. Despite this heterogeneity—and despite not being able to control for successional stage—the effect of pH was clearly observed. We suggest that better controlling for potentially confounding factors (e.g., successional stage) may reveal an even stronger influence of pH.

**Table 1:** Summary of the datasets included in the meta-analysis

Dataset	Description	Successional age range and number of samples (*N*)	Soil pH range	Primer pair and targeted variable region	Sequencing platform and average length of sequencing reads (bp)	Rarefied sequence depth	Total number of OTUs observed	Data source
Systematically sampled across short-term succession	Austre Lovénbreen glacier chronosequence, Svalbard, Norway (AL)	2–142 years (*N* = 38)	6.5–8.0	27F/519R (V1–V3)	454-Pyrosequencing (472-bp)	438	2922	Kim et al. [22]
	Midtre Lovénbreen glacier chronosequence, Svalbard, Norway (ML)	2–87 years (*N* = 39)	7.8–9.4	Bakt_341F/ Bakt_805R (V3–V4)	Illumina Miseq (241-bp)	7148	15,075	This study
	Damma glacier chronosequence, Switzerland (DM)	10–110 years (*N* = 33)	4.8–6.2	27F/519R (V1–V3)	454-Pyrosequencing (261-bp)	3840	12,053	Rime et al. [24]
	Easton glacier chronosequence, Washington, USA (ES)	0–80 years (*N* = 13)	3.9–5.6	27F/338R (V1–V2)	454-Pyrosequencing (242-bp)	95	1118	Castle et al. [23]
Systematically sampled across long-term succession	Wilderness Park sand-dune soil chronosequence, Michigan, USA (SD)	0–4010 years (*N* = 85)	3.1–8.1	27F − YM + 3/ 515R-NK (V1–V3)	454-Pyrosequencing (162-bp)	485	4750	Williams et al. [27]
	Franz Josef Glacier chronosequence, South Island, New Zealand (FJ)	10–120,000 years (*N* = 42)	3.7–7.1	27F − YM + 3/ 515R-NK (V1–V3)	454-Pyrosequencing (228-bp)	576	2035	Jangid et al. [26]
Sampled without regard for successional age	Global scale sampling of soil across a range of biomes	— (*N* = 130)	3.6–8.9	27 F/338 R (V1–V2)	454-Pyrosequencing (213-bp)	514	13,950	Lauber et al. [31] and Chu et al. [29]

### DNA extraction and sequencing

The MoBio Power Soil DNA extraction kit (MoBio Laboratories, Carlsbad, CA, USA) was used to extract the DNA from samples collected along the ML chronosequence. The extracted DNA samples were sent to Macrogen Incorporated (Seoul, Korea) for sequencing. The V3 and V4 region of the 16S rRNA gene was amplified using bacterial primers Bakt_341F and Bakt_805R [35]. The resulting amplicons were sequenced using 300-bp pair-end Illumina MiSeq system (Illumina, San Diego, CA, USA). The 16S rRNA gene sequence data from the ML chronosequence was deposited in the MG-RAST server under project ID mgp21131 (http://metagenomics.anl.gov/linkin.cgi?project=mgp21131).

### Sequence processing

The sequence datasets were analyzed separately as these datasets contained sequences from different variable 16S rRNA gene regions (Table 1). The initial quality filtering steps were different for pyrosequencing and Illumina datasets. All the pyrosequenced datasets, except ES and SD, were quality filtered in Mothur [36]. Briefly, sequences with barcode ambiguities, with read length <150 bp, and with average quality score <25 were removed. The obtained sequences of ES and SD sites were already quality filtered by authors (for detailed information on quality filtering, see refs. [23, 27]). The Illumina sequences from the ML site were quality filtered by following the error correction strategy of Schirmer et al. [37]. Briefly, the paired-end sequences were quality trimmed (Sickle) and error corrected (BayesHammer) before being assembled using PANDAseq [38].

The quality filtered sequences were aligned against SILVA alignment version 123 (http://www.arb-silva.de/). Chimeric sequences were detected and removed via the Chimera UCHIME algorithm in de novo mode contained within mothur [39]. The operational taxonomic units (OTUs) were clustered using the average neighbor clustering algorithm with a threshold of 97% sequence similarity. Phylogenetic null models can be sensitive to sequencing errors, so to avoid spurious results due to sequencing errors, singleton OTUs were not used in subsequent analyses. Finally, each OTU table was rarefied to equal sequence depth (Table 1) by random subsampling to minimize the effect of sequence depth variations among samples.

### Phylogenetic analysis

Aligned sequences of representative OTUs were used to construct a maximum-likelihood tree in FastTree [40]. To test for phylogenetic signal, we first calculated environmental optima for all OTUs with respect to soil pH by following the procedure described by Stegen et al. [2]. Briefly, for each OTU its relative abundance-weighted mean value was calculated for soil pH. To calculate the abundance-weighted mean for a given OTU, we first found all samples in which that OTU was present. We then found the abundance-weighted mean pH of all those samples. To do so, in the calculation of mean pH we weighted each pH value by the abundance of the OTU in the associated sample. This procedure was repeated for each OTU, and the resulting value was used as a rough estimate of that OTU’s pH optimum. Then, between-OTU differences in pH optima were calculated as Euclidean distances. Finally, we used Mantel correlograms to measure the correlation coefficients between differences in pH optima and phylogenetic distances [3, 9], and significance of these correlations was assessed using 999 permutations with Bonferroni correction. We also used Huisman–Olff–Fresco (HOF) hierarchic regression models [41] implemented in the R package “eHOF” [42] as an alternative method to calculate niche optima. The eHOF approach selects the best-fit out of the pre-determined model types (seven types of hierarchical models) for each OTU, using Akaike information criterion and bootstrapping to stabilize the model choice. We excluded OTUs with type I model fit, because type I model is flat and has no niche optimum.

To evaluate the phylogenetic community assembly, we calculated the standardized effect size measure of the mean nearest taxon distance (SES.MNTD) using the null model “taxa.labels” (999 randomization) in “picante” R package [43]. Lower values of SES.MNTD (<0) indicate phylogenetic clustering (i.e., co-occurring OTUs are more closely related than expected by chance), whereas higher values (>0) indicate phylogenetic over dispersion (i.e., co-occurring OTUs are less closely related than expected by chance) [44].

The pairwise phylogenetic turnover between communities was calculated as the mean nearest taxon distance metric (from here on we refer to this as βMNTD [2, 45] using “comdistnt” function (abundance.weighted = TRUE) from the “picante” R package [43]). Furthermore, to infer community assembly processes, we implemented a previously developed null modeling approach [3, 9, 13, 46]. To do so, we first calculated the β-nearest taxon index (βNTI), which is the difference between observed βMNTD and mean of the null distribution of βMNTD normalized by its standard deviation. βNTI values <−2 indicate significantly less than expected phylogenetic turnover (homogeneous selection) [13], whereas βNTI values >+2 indicate significantly more than expected phylogenetic turnover (variable selection) [13].

If the observed βMNTD values does not significantly deviate from the null βMNTD distribution (|βNTI| < 2), this indicates that the observed difference in phylogenetic community composition is not the result of deterministic selection [47], and hence it should be due to dispersal limitation (very low rates of dispersal), homogenizing dispersal (very high rates of dispersal), or is not the result of a single dominant process (i.e., it is “undominated”). To differentiate between these scenarios, we further calculated the Bray–Curtis-based Raup–Crick metric (RC_bray_) as described by Stegen et al. [9] on pairwise comparisons with |βNTI| < 2.

The relative contributions of different community assembly processes were estimated following the method originally proposed in Stegen et al. [9] and modified in Stegen et al. [46] and Dini-Andreote et al. [13]. More specifically, the relative contributions of variable and homogeneous selection were estimated as the percentage of pairwise βNTI values that fell above +2 and below −2, respectively. The relative contribution of dispersal limitation was estimated as the percentage of pairwise comparisons with |βNTI| < 2 and RC_bray_ > +0.95. The relative contribution of homogenizing dispersal was estimated as the percentage of pairwise comparisons with |βNTI| < 2 and RC_bray_ <−0.95. Pairwise comparisons that did not fall into any of these categories indicate that no single process dominated community assembly. The undominated fraction was therefore estimated as the percentage of pairwise comparisons with |βNTI| < 2 and |RC_bray_| < 0.95. The logic behind this approach and simulation models that support these inferences are provided in Stegen et al. [9, 46] and Dini-Andreote et al. [13].

### Statistical analyses

To test the effect of successional age and soil pH on SES.MNTD across all datasets, we used a generalized additive mixed model (GAMM). GAMM was fitted using the “gamm” function of “mgcv” R package [48]. We used cubic regression spline smoothers for each explanatory variable in the GAMM, with study site as a random factor.

To assess the relative influence of stochastic and deterministic assembly processes across successional soils, we compared all possible pairwise comparisons of βNTI values within different successional ages. To further evaluate the variation in community assembly processes along gradients of soil pH, βNTI values—which are derived from pairwise comparisons—were regressed against Euclidean distance matrices of soil pH. This was done both within and among successional ages. The statistical significance of the resulting comparisons was determined by Mantel tests with 999 permutations. Further, to assess the relationship between phylogenetic turnover and soil pH or spatial distance after controlling for spatial or soil pH distance, we performed partial Mantel test with 999 permutations. Similarly, the influence of soil pH on βNTI was also compared with other environmental variables and spatial distance. These analyses were performed using the “mantel” function of “ecodist” R package [49]. As the library size varied widely between subsampled datasets with lower coverage in some of the datasets (Table 1), we evaluated the effect of library coverage on results by varying the library size and subsampling more and less sequences from datasets with lower and higher library coverage, respectively.

## Results

### Phylogenetic signal

Phylogenetic signal was very similar between the abundance-weighted mean and eHOF approaches. Both methods indicated significant phylogenetic signal across short phylogenetic distances (Figs. S1 and S2). Therefore, we calculated SES.MNTD and βNTI because both of these metrics emphasize phylogenetic relationships across short phylogenetic distances. However, there are some caveats related to the use of eHOF. In eHOF approach, model fits can be poor when data are sparse and/or unevenly distributed across the environmental axis, and many OTUs can be removed if the best-fit models are commonly type I, which is flat and has no niche optimum.

### Trends in phylogenetic community assembly

**Fig. 1:**
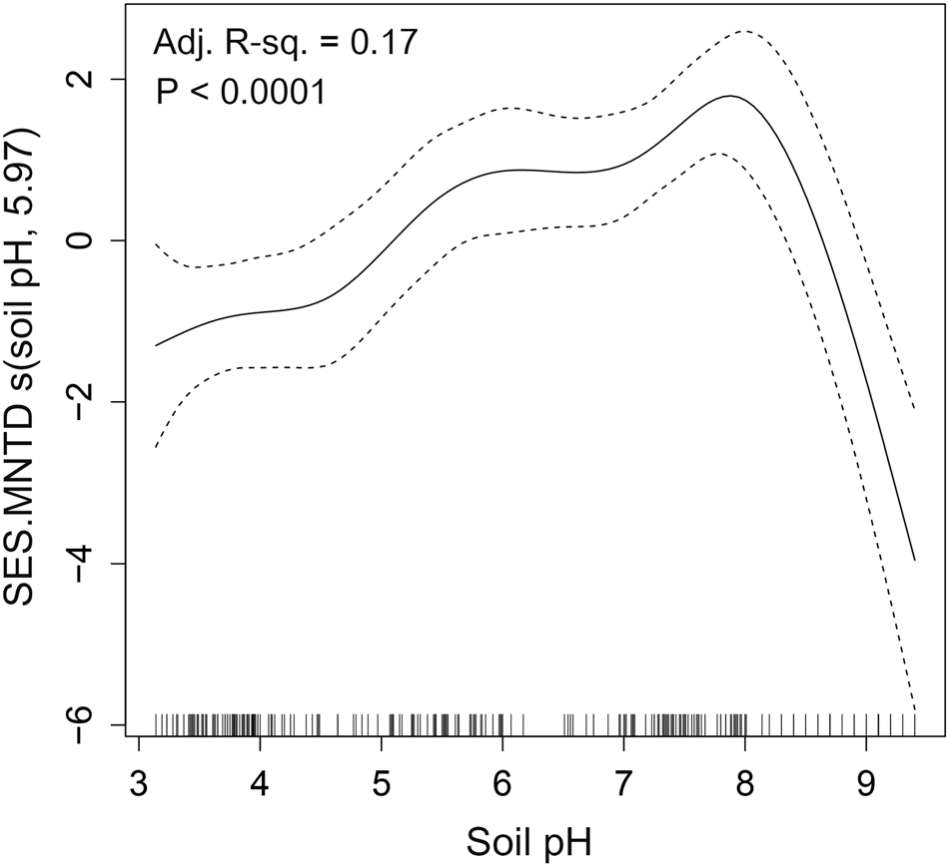
Effect of soil pH on SES.MNTD of bacterial communities (solid line) across all datasets obtained from generalized additive mixed model (GAMM). The *y*-axis shows the contribution of the fitted centered smooth terms (soil pH, estimated degrees of freedom) to SES.MNTD. Ticks along the *x*-axis indicate the distribution of data for soil pH. The dotted lines represent the upper and lower 95% confidence intervals

**Fig. 2:**
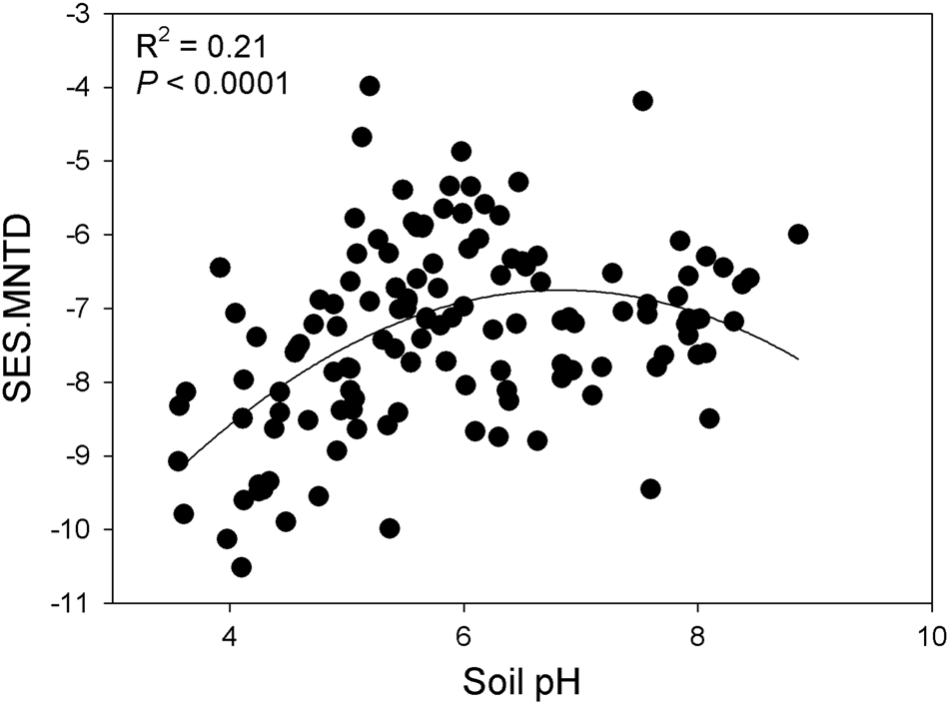
Relationship between soil pH and SES.MNTD of bacteria in samples collected across several different biomes [29, 31]

The mean values of SES.MNTD were significantly less than zero in all datasets (Fig. S3; one sample *t*-test, *P* < 0.05), indicating that in each dataset the bacterial community was more phylogenetically clustered than expected by chance. The GAMM analysis showed that only soil pH was significantly related to SES.MNTD across all sites, and it explained 17.2% of the model deviance with a significant (*P* < 0.05) non-linear fit (Table S1). The effect of successional age on SES.MNTD was non-significant. The SES.MNTD values increased from pH ~3 to ~7.8 and exhibited a sharp decline across pH values >7.8 (Fig. 1). This result indicates that bacterial community assembly was phylogenetically more clustered in more acidic and alkaline soils and phylogenetically less clustered in soils close to neutral pH. As the effect of successional age was not significantly related to SES.MNTD in the datasets that were sampled systematically across successional ages, we further analyzed the SES.MNTD pattern in soils collected globally, and without regard for successional age. These samples spanned a wider range of pH 3.6–8.9 [29, 31]. The SES.MNTD values showed a unimodal pattern along the pH gradient (Fig. 2; Adj. *R*^2^ = 0.21, *P* < 0.0001), peaking at close to neutral pH. This result further supports the importance of soil pH in shaping community assembly processes in soil bacterial communities.

### Relative influence of deterministic and stochastic assembly processes

**Fig. 3:**
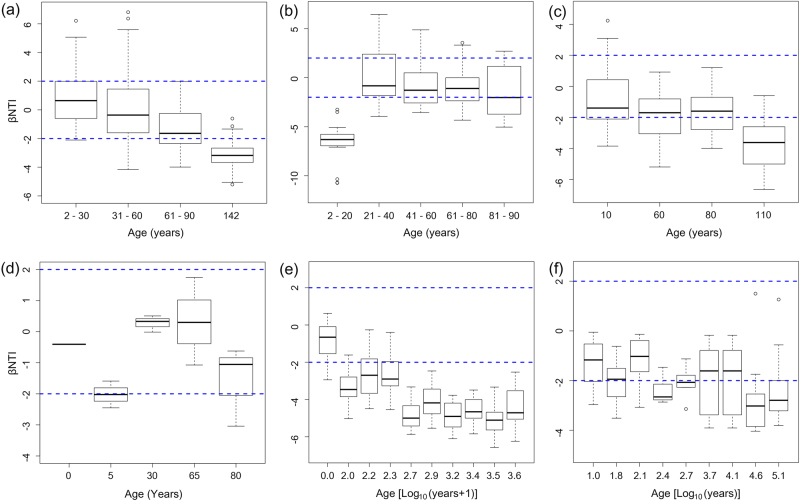
Patterns of βNTI across successional ages in **a** AL, **b** ML, **c** DM, **d** ES, **e** FJ, and **f** SD chronosequences. Horizontal dashed blue lines indicate upper and lower significance thresholds at βNTI = +2 and −2, respectively

We examined the relationship between βNTI and successional age to infer changes in the relative influences of deterministic and stochastic assembly processes along successional chronosequences. The pairwise comparisons of βNTI values within each successional age category indicated various patterns in different datasets (Fig. 3). In AL, DM, FJ, and SD chronosequences, the βNTI distributions gradually shifted over the successional ages, from stochastic community assembly (|βNTI| < 2) to homogeneous selection (βNTI <−2) (Fig. 3). However, the trend in the βNTI distribution over successional ages was opposite in the ML chronosequence, where homogeneous selection was dominant in early successional soils and then shifted towards dominance of stochastic assembly in late successional soils (Fig. 3). In the ES chronosequence, stochastic community assembly remained dominant across all successional ages.

**Fig. 4:**
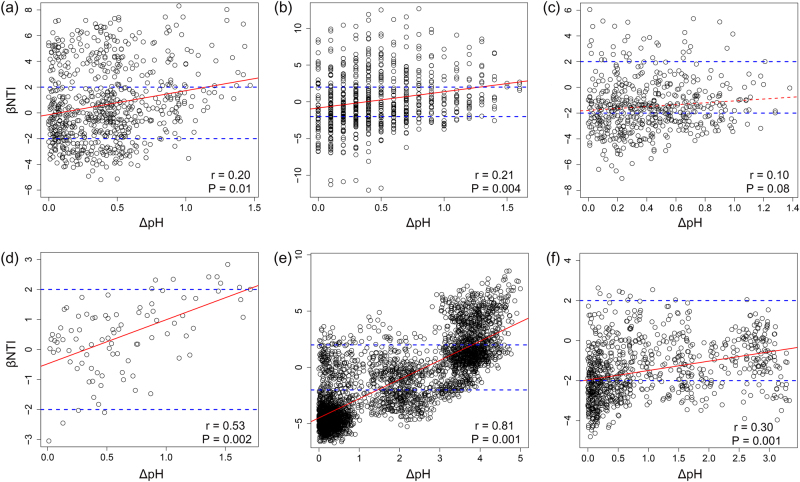
The relationships between βNTI and differences in soil pH for **a** AL, **b** ML, **c** DM, **d** ES, **e** FJ, and **f** SD chronosequences

**Fig. 5:**
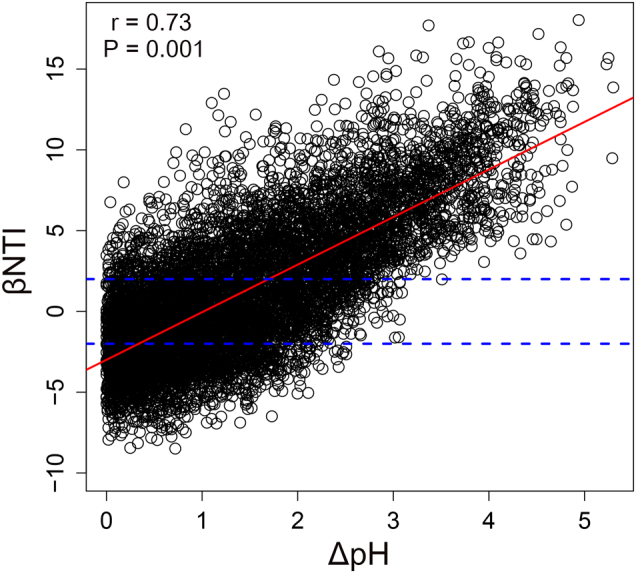
The relationship between βNTI and differences in soil pH for samples collected at global scale across several different biomes [29, 31]

Pairwise comparisons of βNTI values both within and among successional ages were significantly correlated to differences in soil pH (Fig. 4), except in the DM chronosequence. After controlling for spatial distance, soil pH distance was still significantly correlated with βNTI within four sites (Table S2). After controlling for soil pH differences, spatial distances were significantly correlated with βNTI only in AL and DM chronosequences (Table S2). The relationship between soil pH and βNTI remained significant across most of the datasets even when controlled for other measured environmental variables and spatial distance (Table S3). When we combined all possible pairwise comparisons of βNTI values from all datasets, the relationship between βNTI and difference in soil pH remained significant (Fig. S4). These results were further supported in a global scale dataset, where we observed a strong correlation between βNTI and difference in soil pH (Fig. 5). These results indicate that as the difference in soil pH increases, there is a transition in bacterial community assembly processes from homogeneous selection, to stochasticity, to variable selection.

We further divided each successional dataset into different soil pH categories. Within each pH category pairwise comparisons of βNTI showed that the relative influence of homogeneous selection was stronger in highly acidic or alkaline soils (Fig. S5), whereas stochastic community assembly was dominant in soils close to neutral pH (Fig. S5). We found similar results in the global scale dataset (Fig. S6), with dominance of homogeneous selection in more acidic and alkaline soils, and dominance of stochastic assembly in soils close to neutral pH.

### Quantitative estimates of assembly processes

**Fig. 6:**
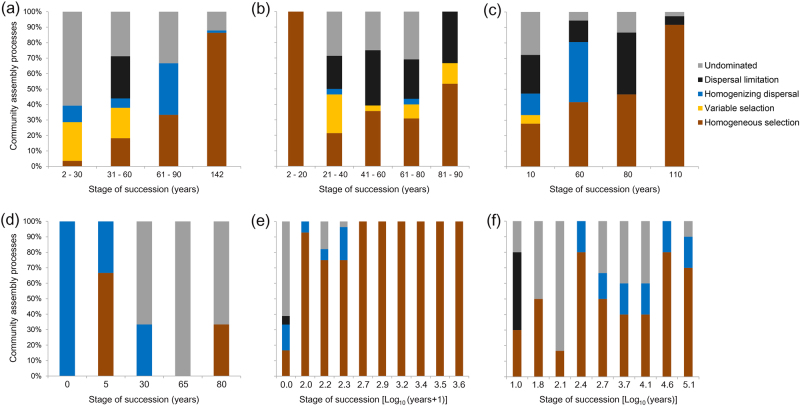
The percent of turnover in bacterial community assembly governed primarily by various deterministic (homogeneous and variable selection) and stochastic processes (dispersal limitation and homogenizing dispersal), as well as the fraction that was not dominated by any single process, across successional ages in **a** AL, **b** ML, **c** DM, **d** ES, **e** FJ, and **f** SD chronosequences

We quantified the relative contributions of each assembly processes over successional ages in all datasets (Fig. 6). The trend in the fraction of homogeneous selection was similar in AL, DM, SD, and FJ chronosequences, which increased with successional age (from ~3 to 30% at earliest successional age to ~70–100% at the latest successional age). Also in these datasets, dispersal limitation (0–50%), homogenizing dispersal (0–16%), and the undominated fraction (~27–61%) all showed significant influences in early successional soils; these influences decreased in late successional soils (dispersal limitation: 0–5%, homogenizing dispersal: 0–20%, and undominated: 0–12%). In the ML chronosequence, the fraction of homogeneous selection decreased from 100% in early successional soils to 53.3% at the late successional soils, whereas the fraction of stochastic assembly increased in late successional soils (dispersal limitation: 21%, homogenizing dispersal: 3%, undominated: 28%,). In the ES chronosequence, the community was primarily influenced by stochastic assembly, where homogenizing dispersal (100%) was dominant in early successional soils and assembly was undominated in late successional soils (66.6%). The fraction of various assembly processes also varied across pH categories in all datasets (Fig. S7), with the fraction of deterministic selection processes (homogeneous and variable selection) highest in acidic and alkaline soils, and the fraction of stochastic processes (dispersal limitation, homogenizing dispersal, and undominated) highest in soils close to neutral pH.

### Effect of library size on inferred assembly processes

The effects of library coverage on inferences related to phylogenetic community assembly processes were evaluated by comparing the results at three different randomly subsampled sequence depths (including the original sequence depth) of each dataset. The results showed that except in ES chronosequence, the SES.MNTD and βNTI trends were robust (Table S4; Fig. S8). It is therefore likely that observed patterns and the associated inferences were not the result of biases introduced by variation in sequencing depth. Owing to low coverage, increasing the sampled library size in ES chronosequence lead to removal of most of the samples from early successional soils (0 and 5 years), which also had lower pH values. The reduced sample size and narrow pH range (after increasing the library size) may have resulted in non-significant trends observed in SES.MNTD and βNTI in ES chronosequence.

## Discussion

To make ecological inferences using phylogenetic turnover requires a phylogenetic signal in the ecological niches of OTUs [45, 50]. We detected significant phylogenetic signal across relatively short phylogenetic distances in all datasets. This suggests that more closely related bacterial taxa have more similar niche preferences related to soil pH [51]. Studying the phylogenetic turnover of closely related organisms can therefore be used to infer the underlying ecological processes in these successional soils [2]. These findings are in agreement with other studies, which found significant phylogenetic signal across relatively short phylogenetic distances across a broad range of ecosystems [2, 3, 9, 13].

Successional age was previously suggested as a primary factor governing bacterial community assembly along a glacial chronosequence [52]. It has been hypothesized that niches are spatially distributed over successional ages with more niches becoming available as succession proceeds, which may lead to a temporal transition from deterministic to stochastic assembly. On the other hand, it has also been hypothesized that niches fill through time, leading to a temporal transition from stochastic to deterministic assembly [13]. However, we found that successional age did not influence community assembly processes, which were instead primarily associated with soil pH. We found that extreme soil pH acts as a stringent environmental filter and leads to phylogenetic clustering, whereas the level of clustering is diminished under moderate pH regardless of the successional age. The relationship between soil pH and bacterial community assembly was also evident across a broad range of biomes in which successional age was not controlled (i.e., in the global dataset) [29, 31], which further supports our findings. Environmental filtering is a key determinant of community assembly [44], and this has been shown to result in phylogenetic clustering in bacterial communities [53]. These findings suggest that the relative influences of stochastic and deterministic community assembly processes can vary with successional age primarily because soil pH can vary with age.

Our results indicate that during succession the temporal trajectory of the stochastic-deterministic balance is not governed by time per se, but instead by the temporal trajectory of soil pH. Examining the relative influence of stochastic processes (dispersal limitation, homogenizing dispersal, and the undominated fraction) in early successional soils that differed in pH provides further support to this inference. Specifically, pH was close to neutral in the early successional soils of AL, DM, SD, and FJ, and for these sites had relatively strong influences of stochastic assembly processes. The ML site provided the contrasting scenario in which pH was extreme in early successional soils, which was associated with a strong influence of deterministic assembly.

By complementing the within-community analysis (SES.MNTD) with between-community null model analysis using βNTI we further showed that spatial processes (i.e., dispersal) were overwhelmed by deterministic selection imposed primarily by pH. In support of this inference, we found βNTI to be more strongly associated (partial Mantel coefficient) with soil pH than with spatial distance or other environmental distances in four sites (Tables S2 and S3). It is interesting to note that in AL and DM sites, spatial distance was significantly related to βNTI after controlling for soil pH and other environmental differences. Although traditional analyses that link community dissimilarity metrics (e.g., Bray–Curtis) to spatial distances often infer influences of spatial processes, it has been argued conceptually and shown via simulation that spatial variation in βNTI should not be driven by spatial processes [3, 9, 46]. Previous work linking βNTI with spatial variables has therefore interpreted significant relationships between βNTI and spatial distance as indicating that there are unmeasured, spatially autocorrelated environmental variables driving community composition through a selection-based mechanism, as opposed to dispersal [9]. This is because the variation in the magnitude of βNTI is driven primarily by variation in deterministic processes, not by organismal dispersal [46, 47]. The reason is that the βNTI null modeling approach is designed to detect differences in community composition that arise due to selection on organismal environmental optima, and is therefore not strongly influenced by dispersal-based community assembly. We therefore infer that ecological selection in the AL and DM sites is at least partially governed by environmental variables that were not measured, but that are spatially autocorrelated. It would be interesting in future efforts to attempt to identify these influential, yet unmeasured, variables.

Though soil pH is known to affect bacterial community composition and diversity at local [32, 54], regional [29, 30, 33], and global scales [55], it has not been clear how soil pH effects community assembly processes across these scales. Our results were consistent in both short-term and long-term successional datasets, and even in a heterogeneous global dataset in which successional age could not be controlled for [29, 31]. Our analyses spanned local to global scales and short- to long-term successional trajectories. The results showed consistent patterns across these spatial and temporal scales, indicating that soil pH mediates the relative influences of stochastic and deterministic processes across scales and across a broad range of ecosystems. Our results are conceptually consistent with the findings of Chase [56] on macro-organisms, which demonstrated that the presence of an extreme environmental filter such as drought results in strong deterministic selection. This finding has been further supported by other studies on macro-organisms [57, 58] and micro-organisms [59]. Combining those previous studies with the results observed here indicates that the same ecological principles govern community assembly processes across spatiotemporal scales, ecosystems, and taxonomic groups.

It is also interesting to consider whether there might be some link between the greater role of stochasticity in neutral pH soils and their greater diversity [29, 31, 33]. In an earlier study, Tripathi et al. [33] speculated the existence of a one-way evolutionary filter along pH gradients, whereby lineages of bacteria can easily branch from a more extreme pH environment to colonize—in evolutionary terms—a more neutral pH environment. This will tend to cause adapted lineages to accumulate in neutral pH environments, resulting in their greater diversity. The greater role of stochasticity, with weaker niche-based exclusion in a neutral pH environment, may assist the evolutionary arrival of lineages from more extreme pH environments.

## Conclusions and implications

**Fig. 7:**
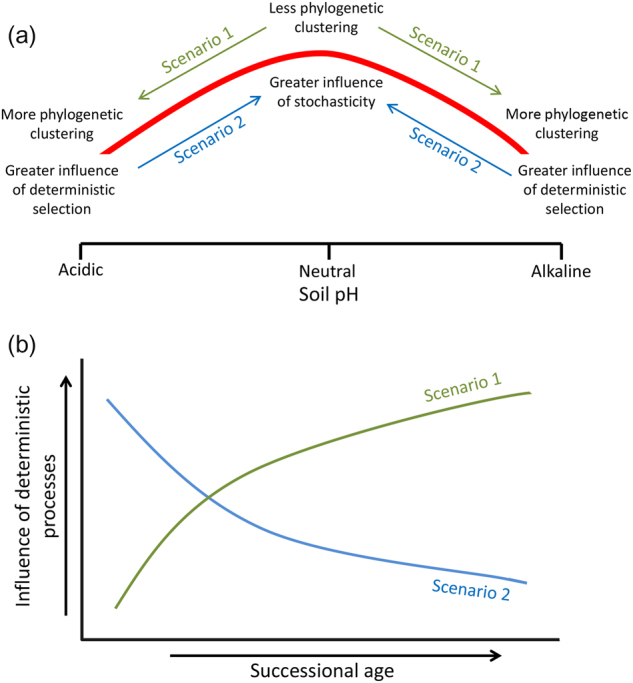
Conceptual model showing two different possible scenarios in bacterial community assembly processes along successional soils with change in **a** soil pH, and **b** temporal trajectory of the influence of deterministic processes for both scenarios

These results together with the previous conceptual model [13] lead us to propose a modified paradigm (Fig. 7), which describes how bacterial community assembly processes differ in relation to pH, across successional soils. We hypothesize two possible scenarios for changes during succession. First, near-neutral pH in early successional soils will lead to more stochastic assembly and phylogenetically less clustered bacterial communities, and a shift in pH towards relatively extreme conditions in late successional soils will lead to more deterministic assembly and phylogenetically more clustered bacterial communities. As an alternative, extreme pH in early successional soils will lead to deterministic assembly and phylogenetically more clustered communities, and progressive shifts in pH toward neutral conditions will lead to weaker selection, more stochasticity and phylogenetically less clustered bacterial communities. This conceptual model coupled with previous models provides a framework that could be experimentally tested in other successional environments.

Our broad examination of soils from around the world reinforces the view that the importance of deterministic vs. stochastic assembly of soil bacterial communities is significantly influenced by soil pH. Thus, microbial communities in different types of soils are influenced by different, pH-influenced balances between stochastic and deterministic community assembly processes. This variation in assembly processes may have implications for ecosystem function [60]. For example in neutral pH soils, biogeochemical function of the soil environment may be less predictable through time and perhaps more spatially variable due to the greater influence of stochasticity. In contrast, the biogeochemical function of soils with more extreme pH may be more consistent through time and more spatially homogeneous. Given the importance of soil processes for overall ecosystem function, this potential linkage between-community assembly processes and soil processes should be evaluated both theoretically and experimentally across a broad range of systems. Nevertheless, although the relationship between pH and assembly processes is clear, it is important to bear in mind that pH accounts for a fairly small proportion (17%) of total variation. Revealing additional factors that influence the balance between stochastic and deterministic assembly processes is an important topic for further investigations.

### Data accessibility

The 16S rRNA gene sequence data of Midtre Lovénbreen Glacier chronosequence samples are deposited in the MG-RAST server under project ID mgp21131 (http://metagenomics.anl.gov/linkin.cgi?project=mgp21131). The R codes used for calculating SES.MNTD and βNTI metrics are provided in the supplemental material.

## Electronic supplementary material

Supplementary tables and figures

Supplementary R codes
