# Supplementary material for: Soil pH mediates the balance between stochastic and deterministic assembly of bacteria
Source: ISME J. 2018 Mar 7;12(4):1072–83. doi: 10.1038/s41396-018-0082-4 (PMC5864241; doi:10.1038/s41396-018-0082-4)
Supplement: Supplementary file 1 — Supplementary tables and figures [file 41396_2018_82_MOESM1_ESM.docx]

**Supplementary Information**

**Soil pH mediates the balance between stochastic and deterministic assembly of bacteria**

Binu M. Tripathi^1a^, James C. Stegen^2a^, Mincheol Kim^1^, Dong Ke^3^, Jonathan M. Adams^4*^, and Yoo Kyung Lee^1*^

^1^Korea Polar Research Institute, Incheon 21990, Republic of Korea

^2^Pacific Northwest National Laboratory, 902 Battelle Boulevard, P.O. Box 999, Richland, Washington 99352, USA

^3^Department of Biological Sciences, College of Natural Sciences, Seoul National University, 1 Gwanak-ro, Gwanak-gu, Seoul, 08826, Republic of Korea

^4^School of Water, Energy and Environment, Cranfield University, Cranfield MK43 0AL, UK

^a^These authors equally contributed to the work

***Correspondence:** Yoo Kyung Lee, E-mail: [yklee@kopri.re.kr](mailto:yklee@kopri.re.kr)

Jonathan M. Adams, E-mail: [j.m.adams@cranfield.ac.uk](mailto:j.m.adams@cranfield.ac.uk)

**Table S1.** Generalized Additive Mixed Model fitted to the SES.MNTD data across all sites, reporting intercept and smooth terms.

| **Parametric coefficients** | **Estimate** | **SE** | **t-value** | ***P*-value** |
| --- | --- | --- | --- | --- |
| Intercept | -6.936 | 1.346 | -5.153 | <0.0001 |
| **Smooth term** | **edf** | **Ref. df** | **F-value** | ***P*-value** |
| Successional age (log transformed) | 1.001 | 1.001 | 0.115 | 0.735 |
| Soil pH | 5.981 | 5.981 | 13.08 | <0.0001 |

SE: standard error

edf: effective degree of freedom

Ref. df: Reference degree of freedom

**Table S2.** Partial mantel test results showing relationship between phylogenetic turnover (βNTI) and spatial and soil pH distances after controlling for spatial and soil pH distances.

| **Effect of selected**  **variable on βNTI** | **Controlling**  **for** | **AL** | **ML** | **DM** | **ES** | **SD** | **FJ** |
| --- | --- | --- | --- | --- | --- | --- | --- |
| Spatial distance | Soil pH | **0.24***** | 0.04 | **0.34***** | 0.18 | -0.19 | -0.02 |
| Soil pH | Spatial distance | 0.06 | **0.22**** | -0.06 | **0.21*** | **0.83***** | **0.34***** |

* *P*≤0.05, ** *P*≤0.001, and *** *P*≤0.0001

**Table S3.** Partial mantel test results showing comparison between phylogenetic turnover (βNTI) and one explanatory distance matrix, while holding the other two explanatory distance matrices constant.

| **Effect of selected**  **variable on βNTI** | **Controlling for** | **AL** | **ML** | **DM** | **ES** | **SD** | **FJ** |
| --- | --- | --- | --- | --- | --- | --- | --- |
| Spatial distance | Environmental  distance (excluding pH)  +  Soil pH distance | 0.24** | 0.02 | 0.38*** | 0.13 | -0.20 | -0.01 |
| Environmental distance ^a^  (excluding pH) | Spatial distance  +  Soil pH | 0.04 | 0.11 | -0.21 | 0.20 | 0.53*** | 0.02 |
| Soil pH distance | Spatial distance  +  Environmental  distance (excluding pH) | 0.02 | 0.19** | -0.02 | 0.24* | 0.64*** | 0.29*** |

* *P*≤0.05, ** *P*≤0.001, and *** *P*≤0.0001

^a^ Environmental variables (other than pH) included in the analysis for each data set were:

AL: Temperature, electrical conductivity, soil moisture, total organic carbon (TOC), and total nitrogen (TN)

ML: Sand, silt, clay, TOC, TN, total phosphorus

DM: Sand, silt, clay, TN, total carbon (TC), dissolved organic carbon, dissolved organic nitrogen, NH_4_, NO_3_, SO_4_, PO_4_, Al, Ca, Fe, K, Mg, Mn, Na, and Zn

ES: TOC, TN, microbial biomass carbon, microbial biomass nirogen, NH_4_, NO_3_, and PO_4_

Fran Josef: TC and TN

Sand dune: Ca, K, and Mg

**Table S4.** Partial mantel test results showing comparisons of βNTI matrices (calculated using three different sequence coverage) in each data set with spatial and soil pH distances after controlling for spatial and soil pH distances.

| **Effect of selected**  **variable on βNTI** | **Controlling for** | **AL** | | | **ML** | | | **DM** | | | **ES** | | | **SD** | | | **FJ** | | |
| --- | --- | --- | --- | --- | --- | --- | --- | --- | --- | --- | --- | --- | --- | --- | --- | --- | --- | --- | --- |
| Subsampling depth |  | 438 | 508 | 659 | 7148 | 500 | 1000 | 3840 | 500 | 1000 | 95 | 150 | 291 | 485 | 801 | 1044 | 576 | 816 | 1138 |
| Spatial distance | Soil pH | **0.2**** | **0.2**** | **0.2*** | 0.04 | 0.1 | 0.05 | **0.3***** | **0.3***** | **0.3***** | 0.2 | 0.1 | 0.2 | -0.2 | -0.2 | -0.2 | -0.02 | -0.004 | -0.1 |
| Soil pH | Geographic distance | 0.1 | **0.1*** | **0.1*** | **0.2**** | **0.3***** | **0.2**** | -0.1 | -0.1 | **-0.1** | **0.2*** | -0.1 | -0.04 | **0.8***** | **0.8***** | **0.8***** | **0.3***** | **0.5***** | **0.5***** |

* *P*≤0.05, ** *P*≤0.001, and *** *P*≤0.0001


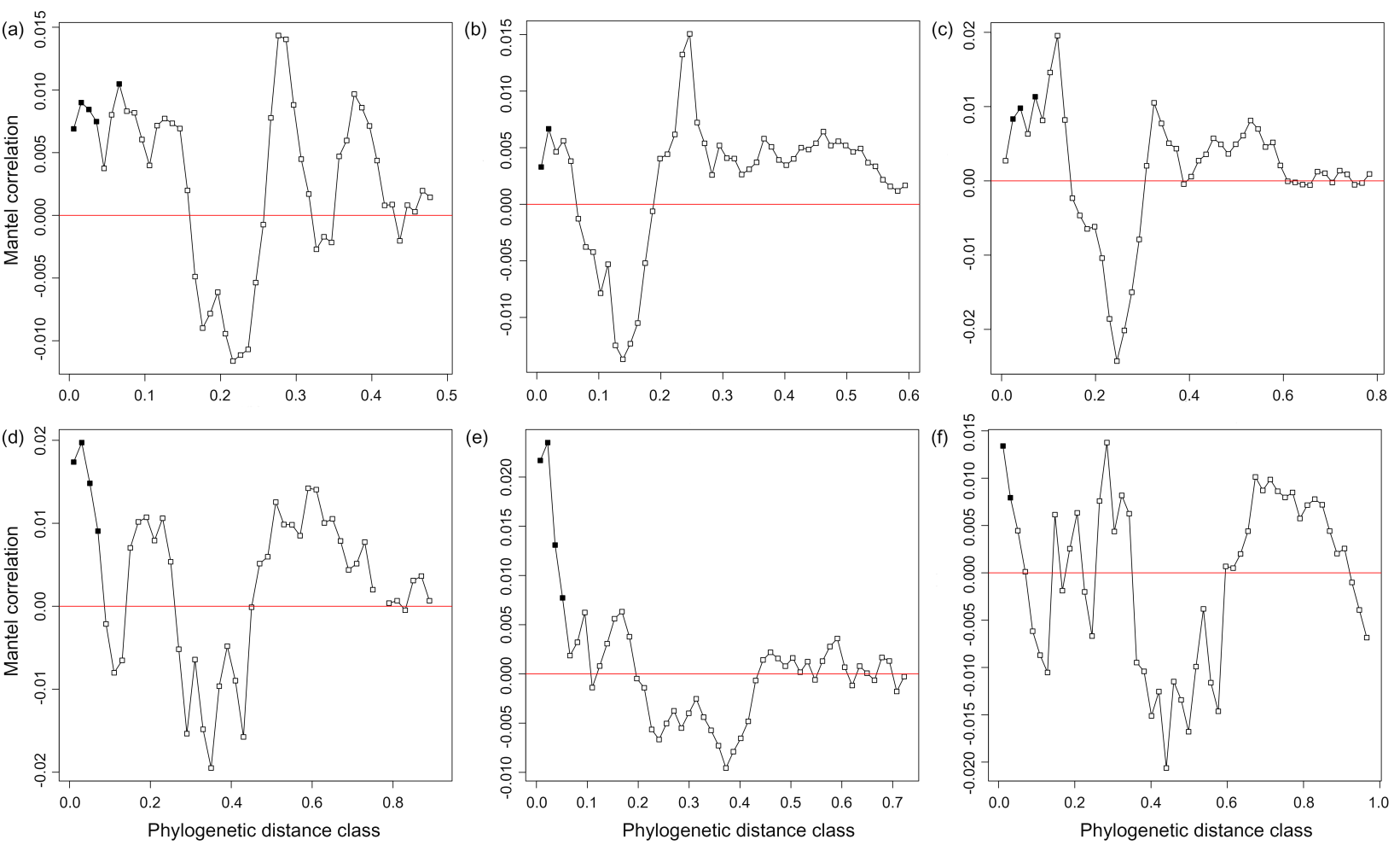


**Fig. S1.** Mantel correlograms between the pairwise matrix of OTU pH (calculated using abundance-weighted mean approach) and phylogenetic distances in (a) AL, (b) ML, (c) DM, (d) ES, (e) SD and (f) FJ chronosequences. Closed squares represent significant phylogenetic signals at the significance level of α<0.05 after Bonferroni correction for multiple testing.

***
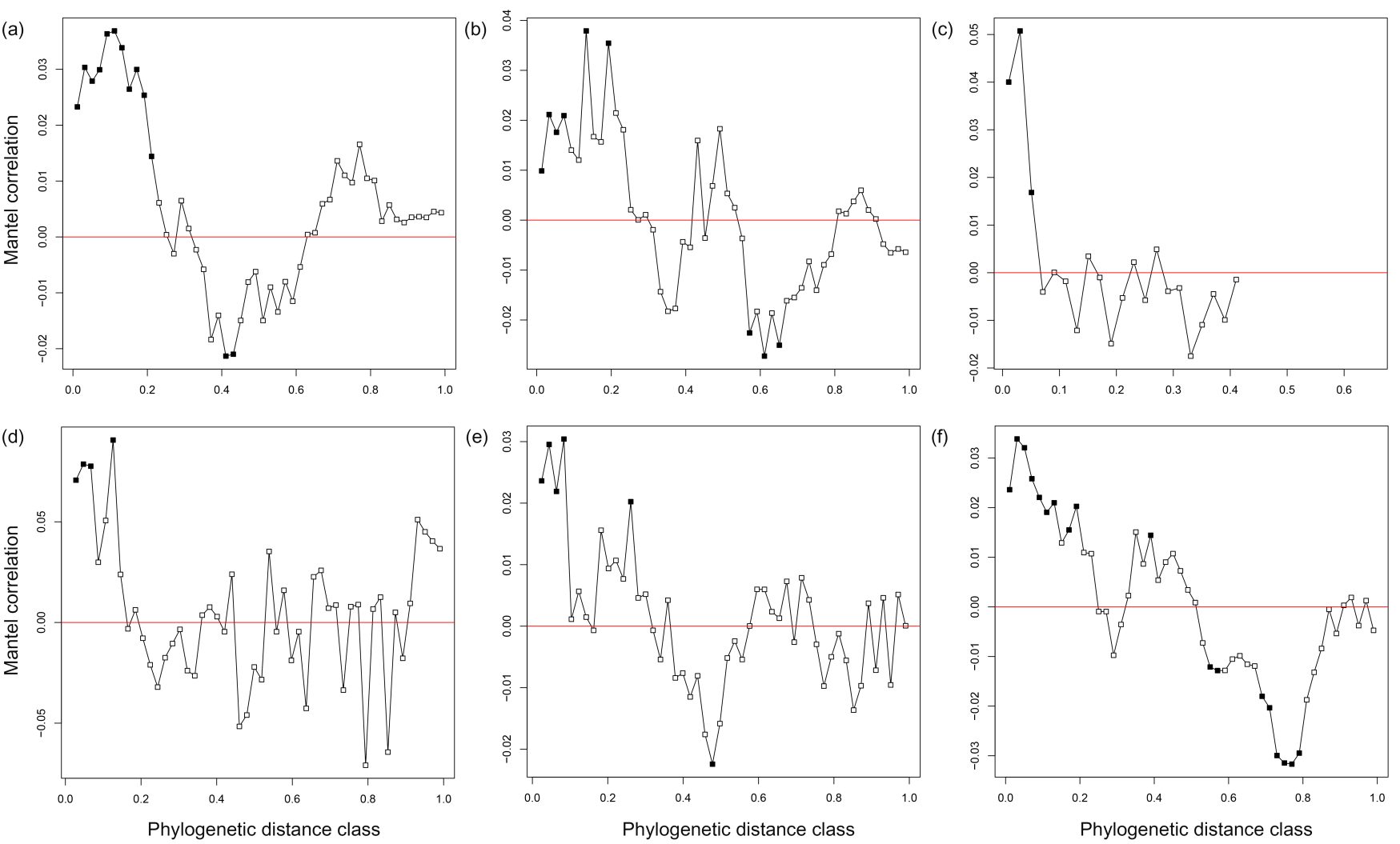
***

**Fig. S2.** Mantel correlograms showing phylogenetic signals between OTU pH (calculated using eHOF approach) and phylogenetic distances in (a) AL, (b) ML, (c) DM, (d) ES, (e) SD and (f) FJ chronosequences.


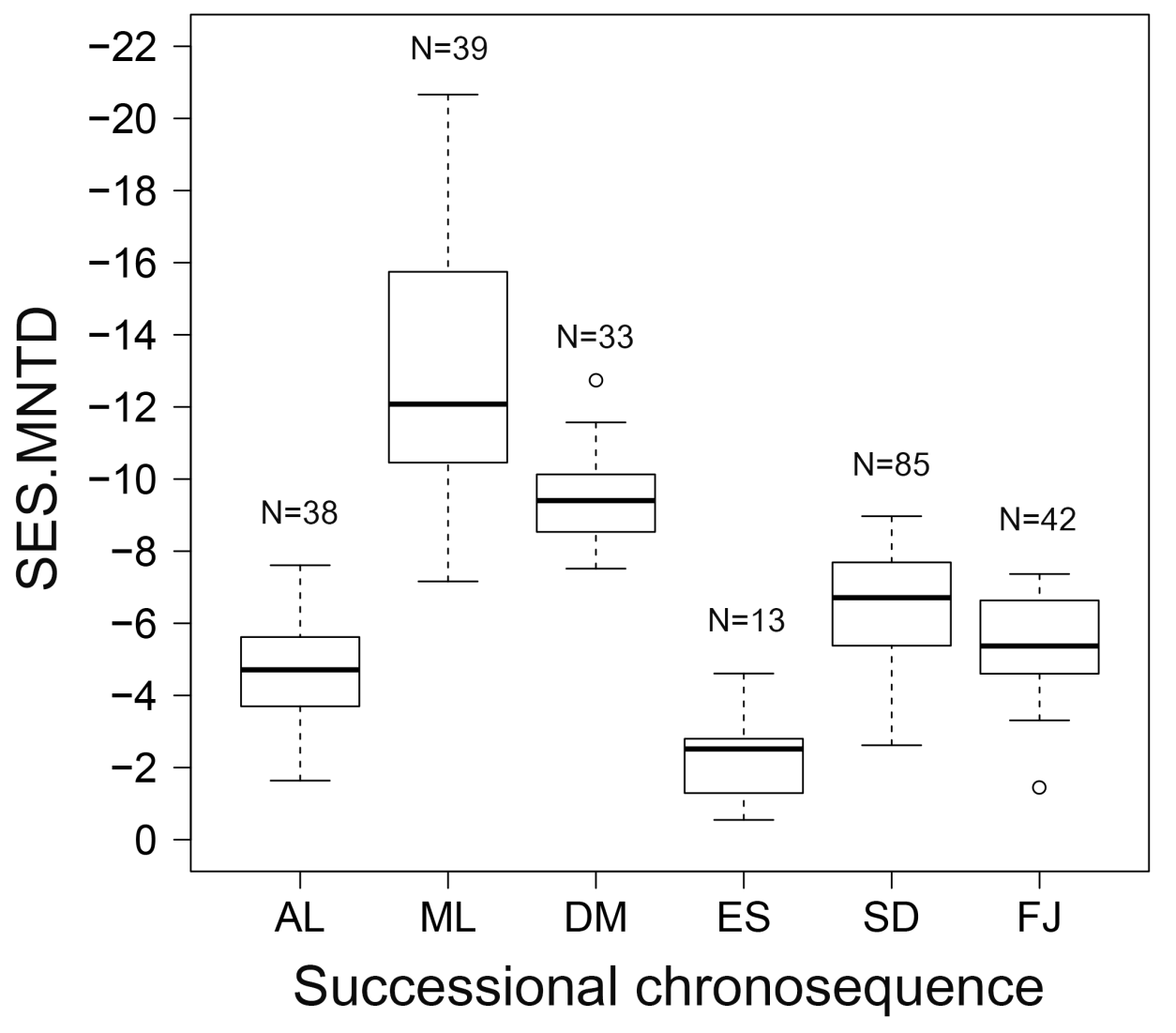


**Fig. S3.** Box plot showing variation in SES.MNTD values across various successional datasets (n = number of samples). In all datasets, the mean values of SES.MNTD were significantly less than zero (one sample t-test, *P* < 0.05).

**
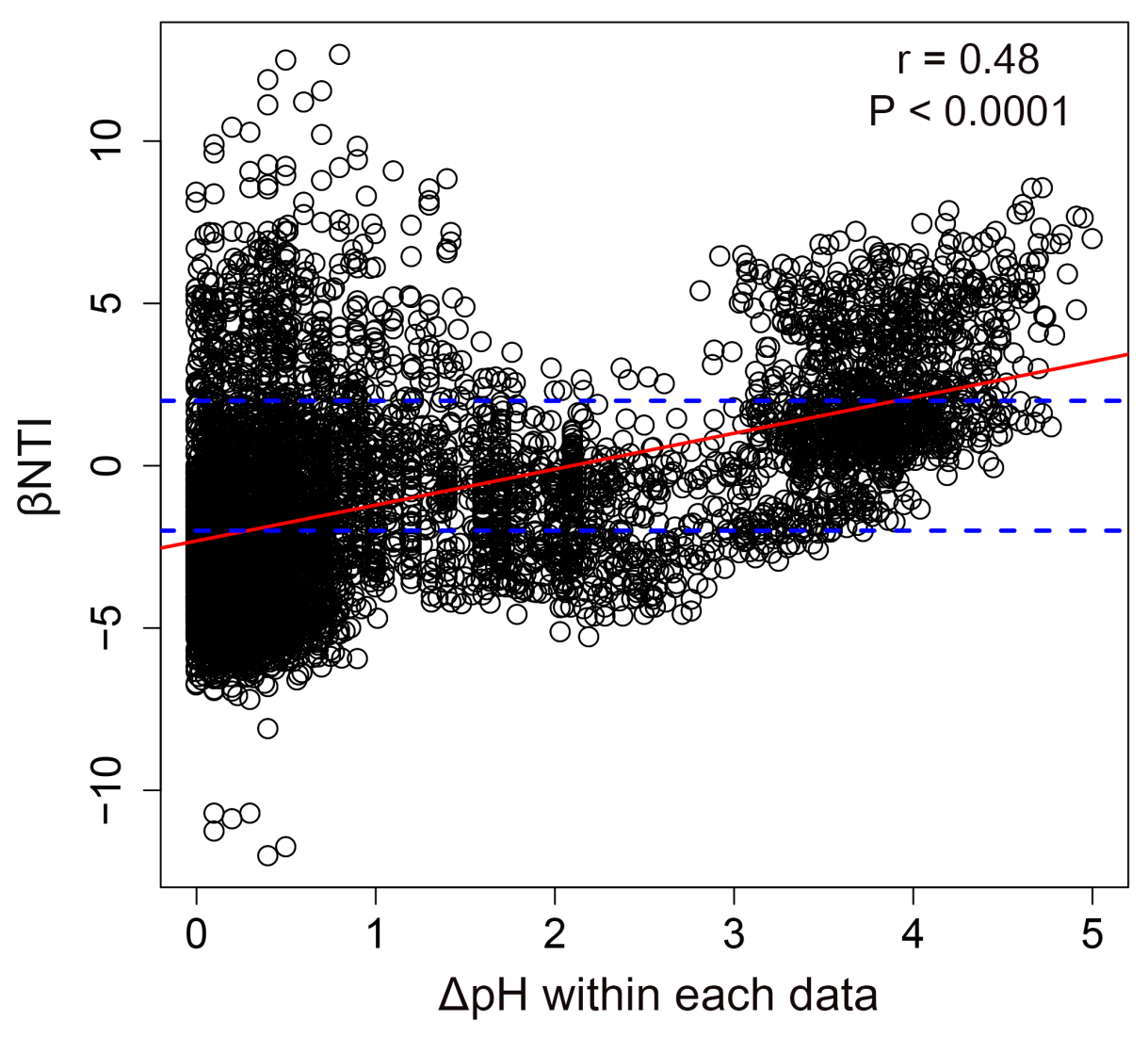
**

**Fig. S4.** The relationship between βNTI and change in soil pH for all successional data sets combined.

**
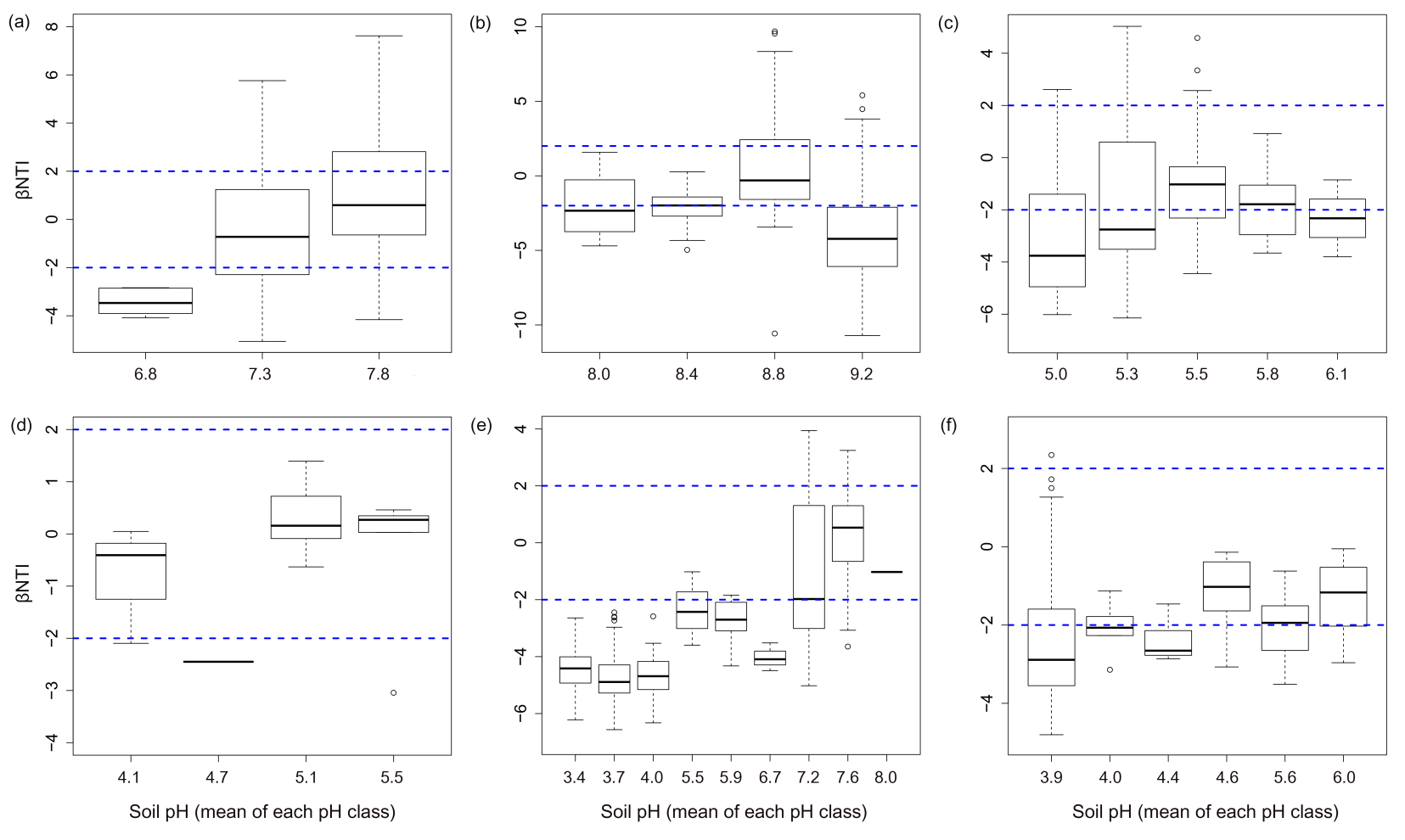
**

**Fig. S5.** Patterns of βNTI across different soil pH categories in (a) AL, (b) ML, (c) DM, (d) ES, (e) FJ and (f) SD chronosequences. Horizontal dashed blue lines indicate upper and lower significance thresholds at βNTI = +2 and −2, respectively.

**
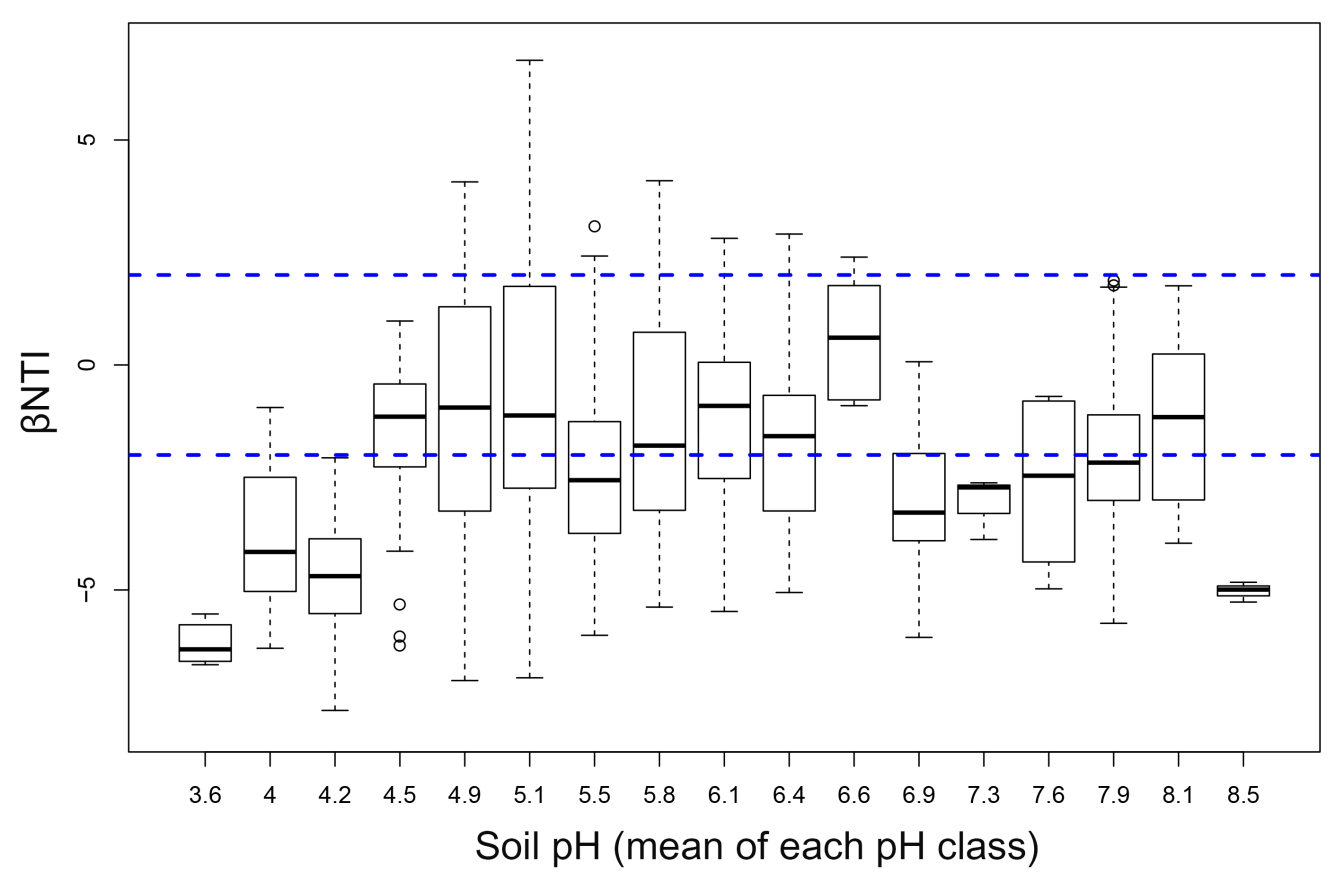
**

**Fig. S6.** Patterns of βNTI across different soil pH categories in samples collected across various biomes.

**
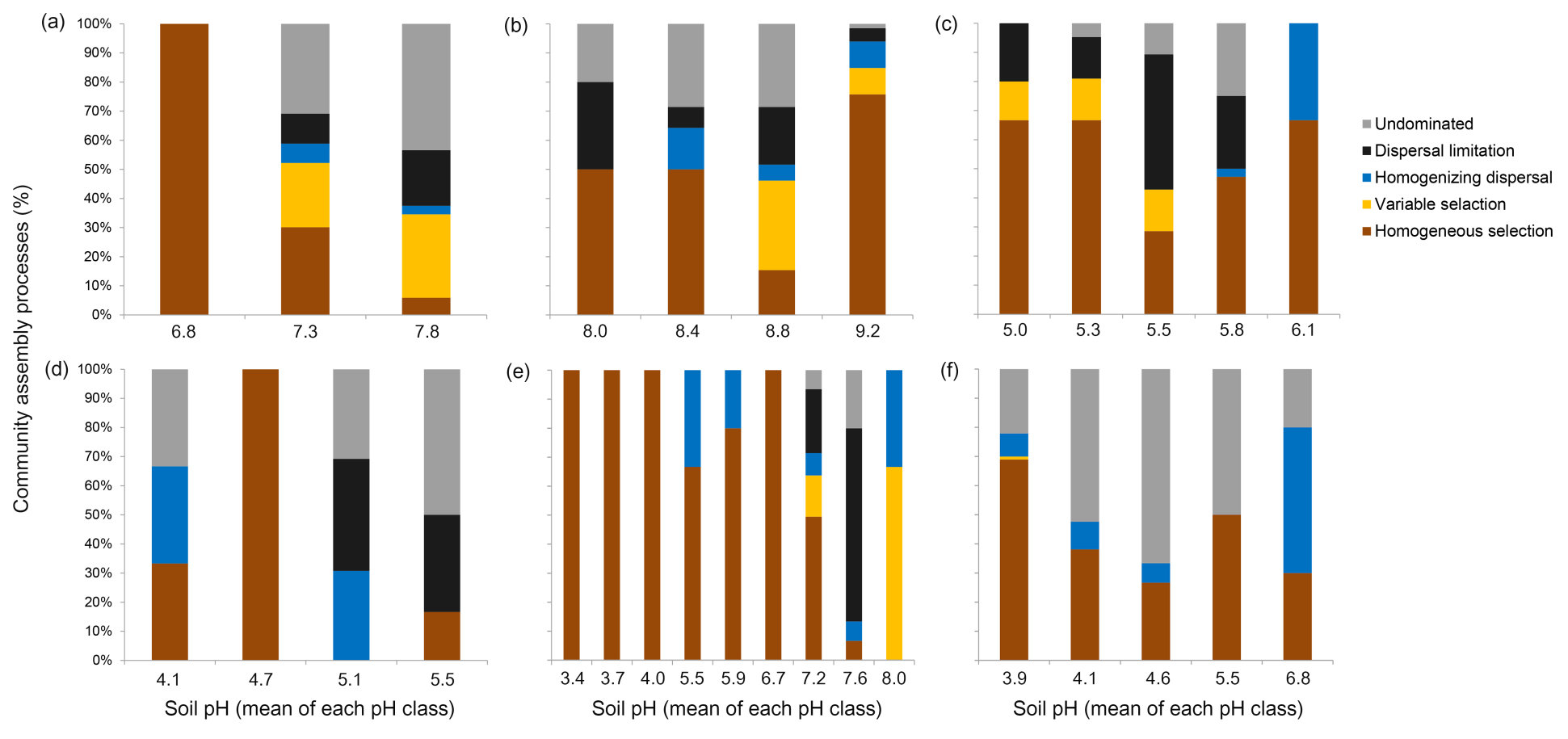
**

**Fig. S7.** The percent of turnover in bacterial community assembly governed primarily by various deterministic (homogenous and variable selection) and stochastic processes (dispersal limitation and homogenizing dispersal), as well as the fraction that was not dominated by any single process, within different soil pH categories in (a) AL, (b) ML, (c) DM, (d) ES, (e) FJ and (f) SD chronosequences.

**
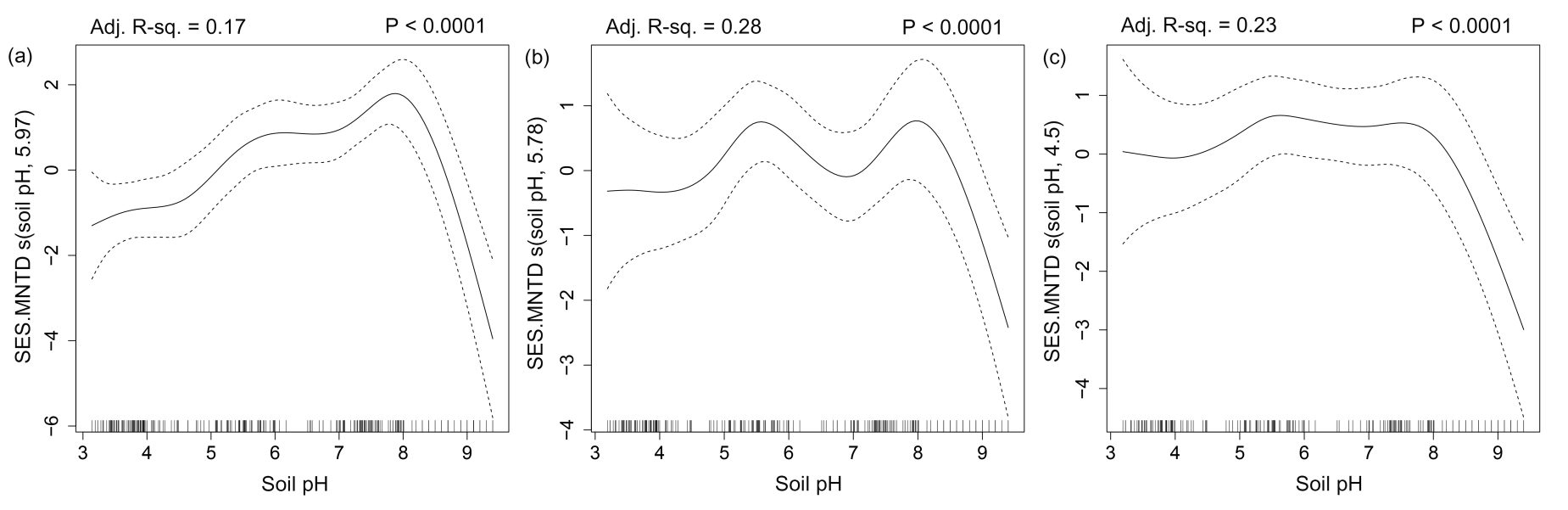
**

**Fig. S8.** Relationships between soil pH and SES.MNTD of bacterial communities across all datasets obtained from Generalized Additive Mixed Model (GAMM) at (a) first (b) second and (c) third subsampling depths (for different subsampling depths please see Table S4).
